# Larval Competition between *Aedes* and *Culex* Mosquitoes Carries over to Higher Arboviral Infection during Their Adult Stage

**DOI:** 10.3390/v16081202

**Published:** 2024-07-26

**Authors:** Adwine Vanslembrouck, Stephanie Jansen, Jacobus De Witte, Corneel Janssens, Stien Vereecken, Michelle Helms, Unchana Lange, Renke Lühken, Jonas Schmidt-Chanasit, Anna Heitmann, Ruth Müller

**Affiliations:** 1 Department of Biomedical Sciences, Institute of Tropical Medicine, Nationalestraat 155, 2000 Antwerp, Belgium; jdewitte@itg.be (J.D.W.); cjanssens@itg.be (C.J.); svereecken@itg.be (S.V.); rmuller@itg.be (R.M.); 2 Department of Biology, University of Antwerp, Groenenborgerlaan 171, 2020 Antwerp, Belgium; 3 Bernhard Nocht Institute for Tropical Medicine, Bernhard-Nocht-Straße 74, 20359 Hamburg, Germany; stephanie.jansen@uni-hamburg.de (S.J.); helms@bnitm.de (M.H.); unchana.lange@bnitm.de (U.L.); luehken@bnitm.de (R.L.); jonassi@gmx.de (J.S.-C.); heitmann@bnitm.de (A.H.); 4 Faculty of Mathematics, Informatics and Natural Sciences, University of Hamburg, Bundesstraße 55, 20146 Hamburg, Germany; 5 Department of Biomedical Sciences, University of Antwerp, Universiteitsplein 1, 2610 Antwerp, Belgium

**Keywords:** *Aedes albopictus*, *Aedes japonicus*, arbovirus, viral RNA titer, chikungunya virus, *Culex pipiens*, infection rate, Japanese encephalitis virus, West Nile virus

## Abstract

The common house mosquito (*Culex pipiens*) is a native vector for West Nile virus (WNV). Invasive species like the tiger mosquito (*Aedes albopictus*) and Asian bush mosquito (*Aedes japonicus*) are rapidly spreading through Europe, posing a major threat as vectors for dengue, chikungunya (CHIKV), and Japanese encephalitis virus (JEV). These mosquitoes share a similar ecological niche as larvae, but the carry-over effects of aquatic larval interactions to the terrestrial adult stage remain largely unknown and their medical relevance requires further investigation. This study examines the context dependency of larval interactions among *Aedes albopictus*, *Aedes japonicus*, and *Culex pipiens*. The survival, development time, growth, and energetic storage were measured in different European populations within density-response (intraspecific) experiments and replacement (interspecific) experiments at 20 °C and 26 °C. Overall, *Ae. japonicus* was the weakest competitor, while competition between *Ae. albopictus* and *Cx. pipiens* varied with temperature. Adults emerging from this larval competition were infected as follows: *Culex pipiens* with WNV, *Ae. albopictus* with CHIKV, and *Ae. japonicus* with JEV. While no JEV infection was observed, mosquitoes experiencing interspecific interactions during their larval stages exhibited higher infection rates and viral RNA titers for CHIKV and WNV. This increased susceptibility to viral infection after larval competition suggests a higher risk of arbovirus transmission in co-occurring populations.

## 1. Introduction

Over the past 50 years, the global incidence of dengue has increased 30-fold [1]. By 2023, more than 100 countries reported the circulation of chikungunya virus (CHIKV), putting an estimated 1.3 billion people at risk of chikungunya fever globally [2]. A growing number of autochthonous dengue cases have been observed in Europe, with 130 cases in 2023 [3]. The first autochthonous chikungunya outbreak in Europe occurred in Italy in 2007. Since then, sporadic outbreaks of chikungunya have been observed, which have often been associated with extreme climate events [4]. In 2017, a total of 270 confirmed and 219 probable autochthonous chikungunya cases were observed [5]. An increase in autochthonous cases of WNV has been recorded, with 1113 cases and 92 deaths in 2022 [6]. These species have a wide distribution, making any knowledge about their viral infection and implications for arbovirus titer relevant for Europe and regions with a high arboviral burden [1].

*Aedes* mosquitoes are the primary vectors of dengue (DENV) and CHIKV [7]. In Europe, these invasive species include the Asian tiger mosquito *Ae. albopictus* (Skuse, 1894), the Asian bush mosquito *Ae. japonicus japonicus* (Theobald, 1901), and the Korean bush mosquito *Ae. koreicus* (Edwards, 1917) [8]. There are four subspecies of *Ae. japonicus*, but only *Ae. japonicus japonicus* is found in Europe [9]; therefore, it is referred to herein as *Ae. japonicus*. *Aedes albopictus* and *Ae. japonicus* are considered to be among the fastest-spreading invasive species [9]. Their spread and establishment to new regions is largely influenced by climate change and international trade [8,10]. Additionally, anthropization of the landscape is a significant factor influencing the dynamics of vector-borne pathogens [1]. Among the main consequences, apart from nuisance due to high abundances, are outbreaks of mosquito-borne diseases. It has been observed that local outbreaks typically manifest within a time frame of 5 to 15 years subsequent to the establishment of *Ae. albopictus* populations [11]. This statement is supported by the recent autochthonous outbreaks of DENV and CHIKV in Europe [12].

Additionally, *Culex pipiens* Linnaeus, 1758, is a known vector for WNV. This species is endemic, widespread, and abundant in Europe [13]. The species consists of two bioforms: *Cx. pipiens pipiens* and *Cx. pipiens molestus* [14]. With the arrival of *Ae. albopictus* (Albania: 1979 and Italy: 1990) [10] and *Ae. japonicus* (Belgium: 2002) [8,15], it now encounters these invaders in shared larval habitats [16]. All three species occur in artificial containers [17,18].

It is hypothesized that interspecific competition among mosquito larvae may enhance their vector competence for arboviruses [19,20,21]. Competitive stress negatively affects mosquito fitness, which in turn has a detrimental impact on the mosquito immune system and physical barriers against viral infection. This carry-over effect of the aquatic larval interactions to the terrestrial adult stage might be important to be considered when estimating the vector competence of arboviral vectors. For example, Alto, Lounibos [19] observed that *Ae. albopictus* females were smaller and had a higher infection rate, viral RNA titer, and dissemination rate of Sindbis virus (SINV) following larval competition with *Ae. aegypti.* Interspecific competition led to more intense competition compared with intraspecific competition. Similarly, Alto, Lounibos [20] discovered an elevated infection and dissemination rate of DENV in *Ae. albopictus* after larval competition with *Ae. aegypti*. In addition, Bevins [21] reported that *Ae. triseriatus* females had increased mortality, larger size, and higher infection and dissemination rates of La Crosse encephalitis (LACV) following larval competition with *Ae. albopictus*.

*Aedes albopictus* has often been found to be a superior competitor compared with *Ae. aegypti*, *Ae. cretinus*, *Ae. japonicus*, and *Cx. pipiens* during the larval stage in the aquatic environment [22,23,24,25,26,27,28,29]. However, other studies suggest that a balanced coexistence between *Cx. pipiens* and *Ae. albopictus* is possible when sufficient food resources are available [17,28,30]. *Aedes japonicus* is expected not to interact strongly with *Cx. pipiens* [31,32]. It is considered a weak larval competitor compared with *Ae. albopictus* [24,33].

Larval density and competition have been found to affect the egg production, body size, energy reserves, and longevity of adult females [34,35,36,37]. Generally, larger females exhibit higher levels of protein, glycogen, and lipid content upon emergence [38]. These energy reserves are important while searching for a suitable host. They show more biting persistence and higher longevity [21] and vector capacity [39]. Additionally, lipids also regulate the immune response [35,39,40]. The innate immune system of mosquitoes consists of various lines of defense mechanics. The epithelium-lined midgut serves as the initial barrier, while hemocytes play a crucial role as cellular components of innate immunity. Additionally, toll and Imd pathways are responsible for the signaling of the production of antimicrobial peptides (AMP), contributing to the humoral defenses of mosquitoes [35]. Lipids facilitate membrane biogenesis at infection sites and in hemocytes [40]. In addition, lipid droplets could potentially serve as an energy source for microflora and have been linked with the activation of toll-like receptors during DENV infection [39]. In *Ae. aegypti* subjected to larval nutrient stress, a decrease in the number of hemocytes was observed; however, enhanced fat-body-derived immune factors, such as AMPs, were found. Furthermore, transcripts of spaetzle, a key regulator of the toll pathway, and certain immune-related genes were less abundant but demonstrated increased expression [35].

Bevins [21] observed larger females of *Ae. triseriatus* after interspecific larval competition with *Ae. albopictus*. These larger females were more susceptible to developing LACV infections compared with females from intraspecific experiments. Larger females of *Ae. albopictus* have more tissue for virus replication, leading to higher viral RNA titers [19]. Controversially, a study on *Ae. triseriatus* females deriving from malnourished larvae revealed smaller females with a thinner basal lamina [41], a membrane that envelops the midgut and hinders virus movement [20]. These females were also associated with higher infection, dissemination, and transmission rates of a bunyavirus. Telang, Qayum [35] observed that basal lamina thickness was not affected by the size of the female. It was noted that certain immune-related genes were less expressed in stressed larvae but exhibited increased expression in females derived from those stressed larvae [35]. These findings suggest that nutritional stress during the larval stage may result in weaker immune responses in adults [35,41,42], which potentially increase their vector competence. However, these smaller females have shorter longevity, and thus vector capacity, which might be too short to complete the extrinsic incubation period [21,36].

Since the impact from increased competition in the larval stage on arboviral infection is uncertain, this study aimed to investigate the carry-over effect of larval interactions. The focus was on interactions between *Cx. pipiens* and *Ae. albopictus* as well as *Ae. japonicus* in recently established populations from Central Europe. This carry-over was analyzed via examining the effect of intra- and interspecific larval competition on arboviral infection of *Ae. albopictus*, *Ae. japonicus*, and *Cx. pipiens* during their adult stage. The effects of synecological patterns at 20 and 26 °C were assessed by collecting data on larval mortality, development time, behavior, pupal size, and the content of energy reserves. Afterwards, an infection experiment was conducted with the medically relevant CHIKV, JEV, and WNV to test whether significant ecological patterns from larval competition would carry over to an increased infection rate and viral RNA titer, potentially elevating the risk of arbovirus transmission.

## 2. Materials and Methods

### 2.1. Larval Competition Study

#### 2.1.1. Mosquito Material

Material from Germany: *Culex pipiens* s.s./*Cx. torrentium* egg rafts were sampled in Frankfurt in September 2021 and June and August 2022. The egg rafts were stored for several days on humid cotton at 10 °C, before they were placed in softened water at 20 °C or 26 °C to hatch. The *Ae. albopictus* strain (20AAlb.DE-KABS.12) originated from egg collections in Achern in September 2020 and was reared at 28 °C with 80% relative humidity and a 16:8 L–D photoperiod.

Material from Belgium: The *Cx. pipiens* biotype *molestus* strain (20CPip.BE-ITMf.6) originated from larval collections in Hove in 2020 and was reared as a colony with overlapping generations, at 23 °C with relative humidity of 80% and a 16:8 L–D photoperiod. The *Ae. japonicus* experiments were executed with field larvae reared from collected eggs. Oviposition traps were placed and collected in Havelange in June and July 2022 and June 2023. The oviposition sticks were stored at 10 °C for up to several months for experimental use. All rearing and competition experiments took place at 20 °C or 26 °C with relative humidity of 80% and a 16:8 L–D photoperiod in climatic cupboards (CPS-P530 Climatic Cabinet, RUMED Rubarth Apparate GmbH, Laatzen, Germany) at the Merian insectary of the Institute of Tropical Medicine (ITM), Antwerp, Belgium.

Country-specific *Culex* bioforms: The objective of this study was to compare *Ae. albopictus* with *Cx. pipiens*, and *Ae. japonicus* with *Cx. pipiens*, each time with *Cx. pipiens* collected from the same ecoregion. In Germany, it was not possible to sustain a colony of the *Cx. pipiens s.s*./*Cx. torrentium* strain; therefore, egg rafts from the field were used. Species identification was verified through performing a multiplex quantitative real-time PCR (qRT-PCR) as described by Rudolf et al. [43], with all specimens used during the infection experiments. A total of 85 *Culex* specimens were tested, of which 90.6% were *Cx. pipiens pipiens*, 8.2% *Cx. torrentium*, and 1.2% hybrid *Cx. pipiens pipiens* × *molestus*. In Belgium, it was possible to sustain a lab colony with one dominant bioform that contained genetically mixed forms with the bioform *pipiens* (not discriminating multiple-generation hybrids and backcrossings, from hereon called hybrids); we refer to this as *Cx. pipiens molestus*. PCR testing was conducted on the used specimens [44]. A total of 182 *Cx. pipiens* s.l. were tested, revealing 71.4% *Cx. pipiens molestus*, 25.3% hybrid *Cx. pipiens pipiens × molestus*, and 3.3% *Cx. pipiens pipiens*.

#### 2.1.2. Larval Competition Experiments

In the density-response experiments, intraspecific interactions were studied at 20 °C and 26 °C, using 3, 5, 15, 30, and 45 specimens in 1 L cups with 600 mL soft water, in triplicate. To identify the number of larvae that resulted in low mortality rates in the interspecific experiments, the percentage of mortality was documented per larval density (Supplementary file S1, Figure S1). In order to test inter- and intraspecific interactions for *Aedes*–*Culex* 30:0, 20:10, 15:15, 10:20, and 0:30 combinations, replacement experiments were conducted in triplicate at 20 °C and 26 °C in 1 L cups with 600 mL soft water. Within 24 h after hatching, first-instar larvae were placed in the experimental set-up. Larvae were fed three times per week with sieved TetraMin (Tetra, Germany). A dose of 0.5 mg food per larvae was provided during the first four feedings. From the fifth feeding onwards, 1 mg of food was provided per larvae up to an overall total of 6 mg per larvae, based on Müller et al. [17,45]. The time of pupation was registered for each experiment to measure development time and mortality. Three pupal growth parameters (area of cephalothorax, length and width of abdomen) were considered to correct for pupal size.

#### 2.1.3. Video Tracking of Behavioral Variables

Fourth-instar larvae from the same batch as the larval competition experiments were maintained at 20 °C and used for behavioral observations using a high-quality video tracking system with digital image recognition. DanioVision hardware and Ethovision XT 15 software were used to track social interactions, activity, and larval behavior such as total distance moved, velocity, and body contact. Larvae were placed in a petri dish with soft water at room temperature and recording started after 30 s to allow acclimatization. Larvae were recorded for two minutes per experiment. Intraspecific experiments were conducted in triplicate for 1, 2, 5, 10, and 18 larvae per species. Interspecific experiments were run in triplicate with *Aedes*–*Culex* ratios of 18:0, 12:6, 9:9, 6:12, and 0:18.

#### 2.1.4. Photometric Assays for Pupal Lipid, Glycogen, and Protein Content

The first five pupae per experiment were stored at −20 °C to test energy storage. The total content of glycogen, lipid, and protein per pupae was analyzed via photometric assays according to Van Handel [46], Van Handel [47], and Bradford [48], respectively, as described by Bock et al. [49]. Pupal growth parameters (area of cephalothorax, length and width of abdomen) were taken. The abdominal width was considered the most robust metric (selection of parameter based on the lowest coefficient of variation, see Supplementary file S5, Table S1) and was used to correct for pupal size. The following equation was used:

(1) $$\mathrm{Corrected}\;\mathrm{energy}\;\mathrm{content}=\frac{total\;energy\;content}{abdominal\;width}\mathrm{per}\mathrm{mosquito}$$

#### 2.1.5. Data Analysis

The relative crowding coefficient (RCC) was used as a measure of competition. An RCC value of 1 showed that both species were equal competitors. Values below or greater than 1 indicated out-competition [50,51]. The RCC values were calculated for the development time, pupal size, pupal energy content, protein content, and larval behavior (total distance moved, velocity, and body contact) of the three species, from the means of three replicate experiments. The formula used was that described by Harper [52] and adapted by Novak et al. [50] and Oberg et al. [51], according to Müller et al. [17], as follows:

(2) RCCspecies A =0.5SpeciesA20:10SpeciesB20:10+SpeciesA15:15SpeciesB15:15+2SpeciesA10:20SpeciesB10:200/3SpeciesA30:00SpeciesB30:00

There is currently no test available to determine whether these RCC results were significantly different [51]; therefore, we conducted two-way ANOVA, Kruskal–Wallis, or Friedman tests on the raw data (see Supplementary files). A two-way ANOVA was conducted to test for significant differences in larval density, species, or species ratio and the interactions of these. Tukey’s multiple comparisons test was used to compare all data. All statistical tests were conducted using Prism (version 10.1.2, GraphPad Software INC., Boston, MA, USA). Statistical significance was defined as *p* < 0.05. The Kolmogorov–Smirnov test and Shapiro–Wilk test were used to test for normality, and homoscedasticity was tested by curve fitting for appropriate weighting of residuals. When assumptions of normality were violated, a Kruskal–Wallis test or Friedman test was performed instead.

### 2.2. Infection Study

#### 2.2.1. Feeding of Infectious Blood to Mosquitoes That Experienced Larval Competition

Selected treatments from previously conducted larval competition experiments were repeatedly conducted at ITM in Belgium and freshly emerged adult mosquitoes were transferred to the Bernhard Nocht Institute for Tropical Medicine (BNITM) in Germany to determine the rate of infection in mosquitoes that experienced larval competition. All experiments at BNITM were performed under BSL-3 conditions. Seven to ten days after eclosion, mosquitoes were anesthetized with carbon dioxide, sorted into vials, and starved for one (*Aedes*) or two (*Culex*) night(s). Infection was performed as described by Heitmann, Jansen [53]. Blood meal containing 50% human blood (expired banked blood), 30% 8% fructose solution, 10% filtrated bovine serum (FBS), and 10% virus stock was offered with final concentrations of 10^7^ plaque-forming units (PFU)/mL for WNV (clade 1a, strain TOS-09, Genbank HM991273/HM641225), 10^6^ PFU/mL for CHIKV (strain CNR 24/2014, supplied by the European Virus Archive Goes Global project), and 10^6^ PFU/mL for JEV (strain SA-14, GenBank accession number EU073992). All virus stocks were propagated using Vero cells (*Chlorocebus sabaeus*; CVCL 0059, obtained from ATCC, Cat# CCL-81). *Culex pipiens* was infected with West Nile virus (WNV), *Ae. albopictus* with chikungunya virus (CHIKV), and *Ae. japonicus* with Japanese encephalitis virus (JEV). Artificial blood meal was offered for *Cx. pipiens* via cotton sticks overnight, and for *Ae. albopictus* and *Ae. japonicus* in two droplets (50 µL each) per vial for 2 h. Fully engorged mosquitoes were sorted and kept at 24 +/− 5 °C (mimicking fluctuating temperatures between day and night), with a relative humidity of 70%, 12:12 L–D photoperiod, and continuous fructose supply. Mosquitoes were kept for WNV and JEV for two weeks and for CHIKV for one week.

#### 2.2.2. Quantification of Infection Rate

In total, 267 *Cx. pipiens*, 57 *Ae. albopictus*, and 47 *Ae. japonicus* were analyzed individually. Afterwards, specimens were homogenized in 500 µL Dulbecco’s modified Eagle medium (DMEM). RNA was extracted using the MagMAX CORE nucleic acid purification kit (Applied Biosystems, Thermo Fisher Scientific Corporation, Waltham, MA, USA). Using RT-qPCR, viral RNA titer was determined (RealStar Chikungunya RT-PCR Kit 2.0 and RealStar WNV RT-PCR 2.0, both from altona Diagnostics, Hamburg, Germany; for JEV, using a QuantiTect Probe RT-PCR Kit, Qiagen, Hilden, Germany, as described by Huber, Jansen [54]. From these results, the infection rate was calculated as follows:

(3) $$\mathrm{Infection}\;\mathrm{rate}\;(\mathrm{IR})=\frac{virus-positive\;mosquito\;bodies}{number\;of\;fed\;mosquitoes}$$

To exclude natural arbovirus infections, ten randomly selected adult mosquitoes per species were tested by pan-orthobunya-, pan-flavivirus-, and pan-alphavirus-PCR, confirming all specimens as negative [55,56,57].

#### 2.2.3. Statistical Analysis

Principal component analysis (PCA) was applied to examine the 14 variables measured during the competition and infection experiments: larval ratio, pupal size (cephalothorax area (CT), abdominal length (AL) and width (AW)), energy (lipid (L), glycogen (G)), protein content (P), mortality, larval development time, behavioral variables (distance moved, velocity, and duration of the body contact), infection rate (IR), and viral RNA titer (BT) and reduced them to two PC axes. Statistical tests were conducted using Prism (version 10.1.2, GraphPad Software INC., Boston, MA, USA).

## 3. Results

### 3.1. The Effect of Interspecific Treatments on 14 Variables

#### 3.1.1. Mortality

Low mortality during the density-response experiments in microhabitats containing 30 larvae confirmed the use of 30 larvae to be suitable for replacement experiments with the three mosquito species (Supplementary file S1, Figure S1).

The larval mortality of the three mosquito species tested in the replacement experiments was low. The mortality of *Cx. pipiens* s.s./*Cx. torrentium* was higher compared with the *Cx. pipiens* bioform *molestus* competitive treatments (Supplementary file S2, Figure S2). The difference between the species and larval densities were tested; however, results were not significant.

#### 3.1.2. Development Time

The factors *species*, *larval ratio* and their *Interaction* were significant for the *Ae. albopictus* × *Cx. pipiens* s.s./*Cx. torrentium* competitive treatments at both test temperatures, with *Ae. albopictus* developing faster than *Cx. pipiens* s.s./*Cx. torrentium* larvae at 26 °C (Supplementary file S3, Figure S3a,c). The *Ae. japonicus* × *Cx. p. molestus* competitive treatments showed significant differences for the factors *species* and *interaction* at 20 °C, at which *Ae. japonicus* developed faster compared with *Cx. p. molestus* (Supplementary file S3, Figure S3b). At 26 °C, the factors *larval ratio* and *interaction* were significantly different, with *Cx. p. molestus* developing much faster during interspecific competition (Supplementary file S3, Figure S3d).

#### 3.1.3. Larval Behavior

Larval behavioral data on total distance moved, velocity, and body contact showed significant differences only for the duration of body contact in the species-competitive treatments, with body contact being avoided when more *Ae. japonicus* were present (Supplementary file S6, Figure S5).

#### 3.1.4. Pupal Size

There were significant differences in pupal size between the species and ratios. Generally, pupae in interspecific competition were significantly larger compared with intraspecific ones. Only *Cx. pipiens* s.s./*Cx. torrentium* showed smaller cephalothorax size in interspecific competition (Supplementary file S4, Figure S4).

#### 3.1.5. Energy and Protein Storage

Lipid storage in the *Ae. albopictus* × *Cx. pipiens* s.s./*Cx. torrentium* competitive treatments was significantly lower at the higher temperature of 26 °C. At 20 °C, lipid content was lower in the intraspecific treatments compared with interspecific treatments. At 26 °C, a slightly higher lipid content was found in *Ae. albopictus*. The *Ae. japonicus* × *Cx. p. molestus* competitive treatments showed a higher lipid content at 26 °C (Supplementary file S7, Figure S6a,b,g,h). The glycogen content was always higher in *Ae. albopictus* compared with *Cx. pipiens* s.s./*Cx. torrentium* and *Ae. japonicus* (Supplementary file S7, Figure S6c,d,i,j). Protein content was highest at 20 °C for both *Ae. albopictus* and *Cx. pipiens* s.s./*Cx. torrentium*; however, no difference was observed in *Ae. japonicus* (Supplementary file S7, Figure S6e,f,k,l).

#### 3.1.6. Relative Crowding Coefficient

**Figure 1:**
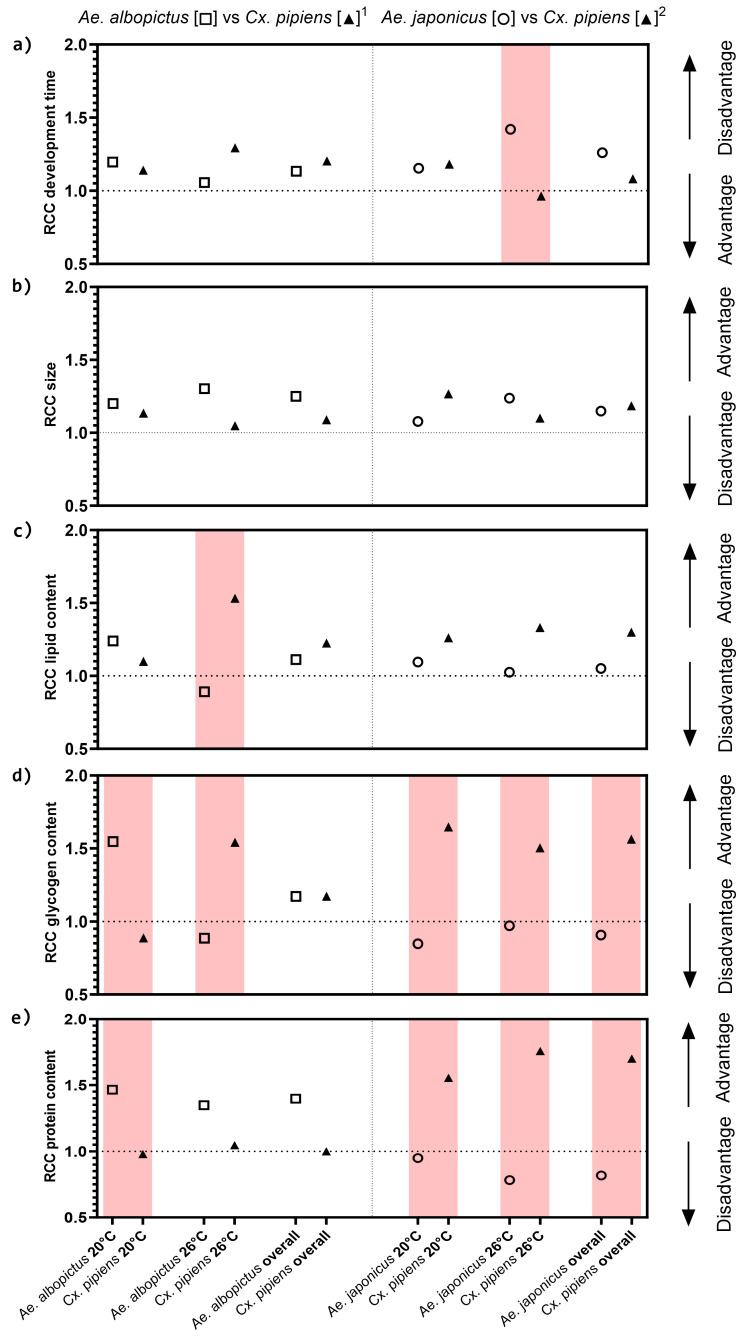
Relative crowding coefficient with advantage or disadvantage. (**a**) Development time for 50% of the pupae to emerge, (**b**) the pupal size, (**c**) lipid, (**d**) glycogen, (**e**) protein content (size-corrected) of combination (1) *Ae. albopictus* vs. *Cx. pipiens* s.s./*Cx. torrentium* and (2) *Ae. japonicus* vs. *Cx. p. molestus* during interspecific competition at 20 °C and 26 °C. In red are the differences, with one of the species having a RCC below 1 indicating out-competition, according to Oberg, Young [51].

For both species combinations, the RCC indicated that the effects of the competition on the larval development were clearest at 26 °C. *Aedes albopictus* had an advantage over *Cx. pipiens* s.s./*Cx. torrentium* at 20 °C, while the opposite was true at 26 °C. *Culex p. molestus* out-competed *Ae. japonicus* at both temperatures (Figure 1a). The RCC for size showed a smaller difference; however, *Ae. albopictus* had a slight advantage over *Cx. pipiens* s.s./*Cx. torrentium.* For the other species combination, *Cx. p. molestus* showed an advantage in terms of larger size at 20 °C and *Ae. japonicus* had an advantage at 26 °C, respectively (Figure 1b).

#### 3.1.7. Synopsis of Synecological Patterns

Our research shows that interspecific competition had a significant impact on all three species, although out-competition was rarely observed among the 14 variables tested. The synecological patterns were predominantly noticeable at the often-overlooked metabolic and behavioral levels. Specifically, *Cx. pipiens* showed pronounced responses at the lower temperature tested, *Ae. albopictus* at the higher temperature tested, and *Ae. japonicus* at both temperatures tested.

**Figure 2:**
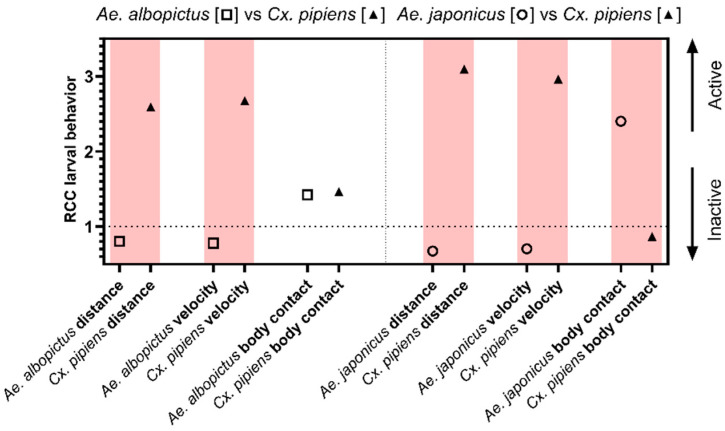
Relative crowding coefficient and its effect on activity or inactivity. Behavior variables: total distance moved, velocity, and duration of body contact for intra- and interspecific larval competition ratios of *Ae. albopictus*, *Cx. pipiens* s.s./*Cx. torrentium*, *Ae. japonicus*, and *Cx. p. molestus*. The differences are indicated in red, with one of the species having a RCC below 1 indicating significant differences in behavior in a competitive environment.

In general, larval (out)competition between species was more apparent in terms of glycogen and protein content rather than development time or pupal size. For the lipid content, an advantage in terms of higher lipid content was seen for *Ae. albopictus* at 20 °C; however, at 26 °C, competition was very clear, with *Cx. pipiens* s.s./*Cx. torrentium* having a negative impact resulting in less lipid storage in *Ae. albopictus*. For *Ae. japonicus*, there was a disadvantage in terms of lipid content compared with *Cx. p. molestus* at both temperatures (Figure 1c). Larval competition clearly influenced the glycogen content of all species under both tested temperature regimes. At 20 °C, *Ae. albopictus* had a competitive advantage over *Cx. pipiens* s.s./*Cx. torrentium* in terms of its higher glycogen content; however, at 26 °C, the opposite was noted with *Ae. albopictus* being negatively affected by *Cx. pipiens* s.s./*Cx. torrentium*. In the other species combination, *Cx. p. molestus* always outcompeted *Ae. japonicus* in terms of glycogen content, resulting in a higher glycogen content in *Cx. p. molestus* in interspecific competition (Figure 1d). For protein content, *Ae. albopictus* had the competitive advantage over *Cx. p. molestus* at both temperatures, with more protein content during interspecific competition. *Aedes japonicus* had a competitive disadvantage at both temperatures compared with *Cx. p. molestus* (Figure 1e). For the larval behavior, both the distance moved and the velocity measured showed more activity for *Aedes* compared with *Culex*. In contrast, *Culex* showed longer body contact compared with *Aedes* (Figure 2).

### 3.2. Infection Study

Infection was successful for *Ae. albopictus* and *Cx. pipiens* s.s./*Cx. torrentium*; however, no infection was found for *Ae. japonicus*. Differences in infection rate and viral RNA titer were found in response to the competition treatment. Assumptions for normality were violated, so a Kruskal–Wallis test was therefore performed on the viral RNA titer data and a Friedman test on the infection rate.

**Figure 3:**
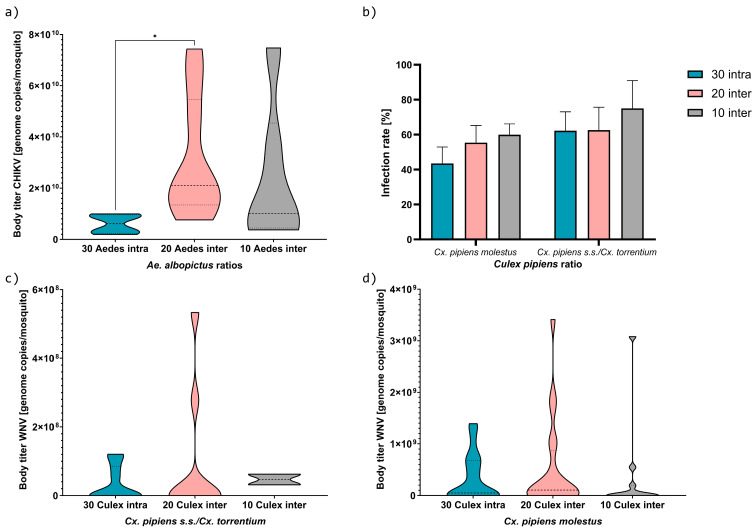
Mean viral RNA body titer per specimen and infection rate per species ratio. (**a**) Mean CHIKV RNA body titer per specimen of *Ae. albopictus*, (**b**) WNV infection rate of both *Cx. pipiens* strains, (**c**) mean WNV RNA body titer per specimen of *Cx. pipiens* s.s./*Cx. torrentium* (see Zenodo repository in data availability statement for separated results for bioforms) from the *Ae. albopictus* combination, (**d**) mean WNV RNA body titer per specimen of *Cx. p. molestus* from the *Ae. japonicus* combination. Infection rates were 100% and 0% for all *Ae. albopictus* and *Ae. japonicus* specimens, respectively; body titer was 0 genome copies per mosquito for *Ae. japonicus*. * = significant difference (*p* = 0.04).

For the *Ae. albopictus* vs. *Cx. pipiens* s.s./*Cx. torrentium* combination, the former species showed a 100% rate of infection with CHIKV. The mean numbers of CHIKV RNA copies per specimen of *Ae. albopictus* were 6.58 × 10^9^, 2.89 × 10^10^, and 1.97 × 10^10^ genome copies for the intraspecific combination, interspecific *Culex*–*Aedes* 10:20, and 20:10 combinations, respectively (Figure 3a). The difference in viral RNA copies per specimen per competitive treatment was significant (Kruskal–Wallis test, *p* = 0.04). The Kruskal–Wallis multiple comparisons test showed a significant difference between *Culex*–*Aedes* 0:30 intraspecific and 10:20 interspecific (*p* = 0.04). The two other comparisons (*Culex*–*Aedes* 0:30 intraspecific vs. 20:10, and 10:20 vs. 20:10 interspecific) were not significantly different (*p* = 0.55 and *p* = 0.69, respectively). *Culex pipiens* s.s./*Cx. torrentium* from this combination had WNV infection rates of 54.8%, 50%, and 66.7% for the intraspecific *Culex*–*Aedes* 30:0 combination and interspecific *Culex*–*Aedes* 20:10 and 10:20 combinations, respectively (Figure 3b). The mean numbers of WNV RNA copies per specimen of *Cx. pipiens* s.s./*Cx. torrentium* were 4.34 × 10^7^, 1.43 × 10^8^, and 5.19 × 10^7^ genome copies for the intraspecific *Culex*–*Aedes* 30:0 combination and interspecific *Culex*–*Aedes* 20:10 and 10:20 combinations, respectively (Figure 3c). Differences in viral RNA titer per competition combination were not significant (Kruskal–Wallis test, *p* = 0.62).

For the *Ae. japonicus* vs. *Cx. p. molestus* combination, no infection with JEV was found for the former species. *Culex p. molestus* had WNV infection rates of 43.9%, 54.7%, and 59.3% for the intraspecific *Culex*–*Aedes* 30:0 combination and interspecific *Culex*–*Aedes* 20:10 and 10:20 combinations, respectively (Figure 3b). None of the infection rates were significantly different (Friedman test, *p* = 0.17). The mean numbers of WNV RNA copies per specimen were 3.90 × 10^8^, 4.42 × 10^8^, and 2.77 × 10^8^ genome copies for the intraspecific *Culex*–*Aedes* 30:0 combination and interspecific *Culex*–*Aedes* 20:10 and 10:20 combinations, respectively (Figure 3d). Differences in the numbers of WNV RNA copies per specimen were not significant between mosquitoes which had previously (not) experienced larval competition (Kruskal–Wallis test, *p* = 0.43).

**Figure 4:**
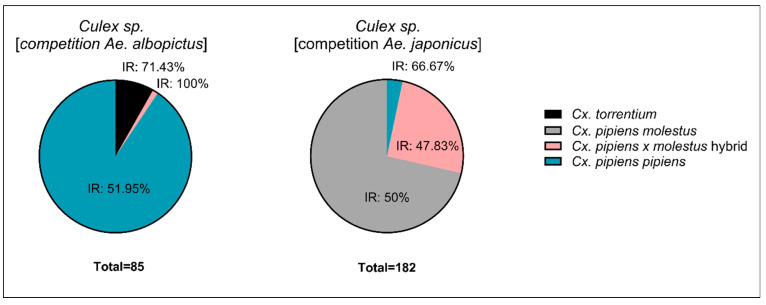
Infection Rates (IRs) of *Culex* species and *Cx. pipiens* biotypes used during the infection study.

For *Cx. p. pipiens*, *Cx. torrentium*, and *Cx. p. pipiens × molestus*, infection rates were 51.95%, 71.43%, and 100%, respectively (Figure 4). The viral RNA titers were 8.1 × 10^7^, 1.4 × 10^8^, and 2.4 × 10^4^ genome copies, respectively. The infection rates were 50%, 47.83%, and 66.67% for *Cx. p. molestus*, *Cx. p. pipiens × molestus*, and *Cx. p. pipiens*, respectively (Figure 4). The viral RNA titers were similar, with 3.99, 3.85, and 3.95 × 10^8^ genome copies.

**Figure 5:**
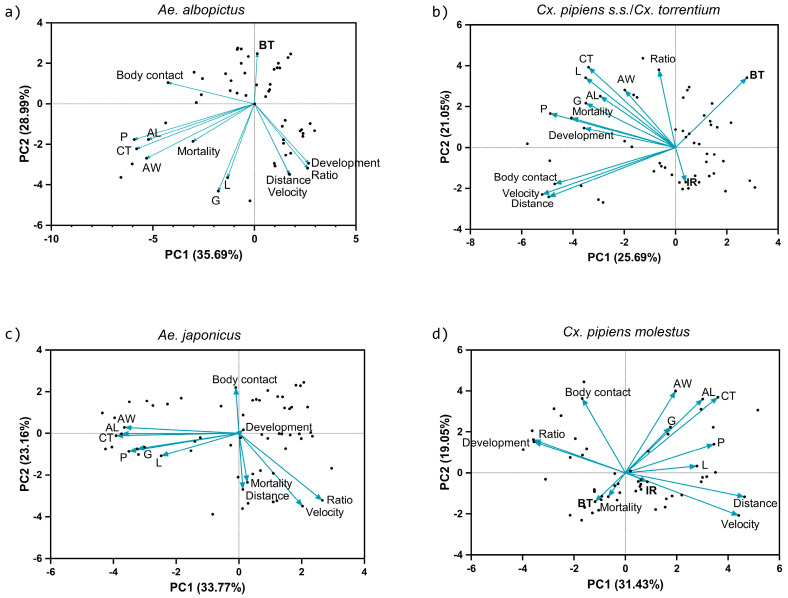
Principal Component Analysis (PCA) of total dataset with variables. Development time, larval ratio, mortality, cephalothorax length (CL), abdominal length (AL), abdominal width (AW), lipid (L), protein (P), glycogen (G), total distance moved, velocity, body contact, viral RNA body titer (BT), and infection rate (IR) for different species competition treatments in (**a**,**b**) *Ae. albopictus* and *Cx. pipiens* s.s./*Cx. torrentium* and (**c**,**d**) *Ae. japonicus* and *Cx. p. molestus* combinations at 26 °C. Infection rate is not included for *Ae. albopictus* and *Ae. japonicus*, viral RNA body titer is not included for *Ae. japonicus*.

The two PC axes explained about 54.71 ± 6.81% of the variation in these 14 variables (Figure 5). Overall, pupal size (AL, AW, CL), energy reserves (L, G) and protein content were positively correlated. Total distance moved and velocity were also grouped together. For *Ae. albopictus*, the PCs explained a total of 64.68% of the proportion of variance (Table 1). Larval ratio and development were correlated and negatively linked with pupal size and protein content; therefore, there was a link on the PC1 axis between interspecific larval ratio, shorter development, larger pupal size, and more protein content. The PC2 axis showed a link between a high viral RNA titer and interspecific competition, low contents of energy storage, and less larval movement (Figure 5a, Table 1).

For the corresponding *Cx. pipiens* s.s./*Cx. torrentium* strain, the PCs explained a total of 46.74% of the proportion of variance (Figure 5). For PC1, the variables velocity, distance moved, protein content, body contact, mortality, and development were correlated, meaning that lower values for these variables related to higher viral RNA titers under interspecific competition. The velocity and distance moved were negatively correlated to cephalothorax size, larval ratio, viral RNA titer, and lipid content in PC2. This indicated that less larval movement contributed to a higher viral RNA titer, but also that larger pupae with more lipid content increased the viral RNA titer. Infection rate was negatively correlated with larval ratio, pupal size, energy reserves, protein content, mortality, and development time (Figure 5b, Table 1). For both *Ae. albopictus* and *Cx. pipiens* s.s./*Cx. torrentium*, the viral RNA titer was higher with interspecific ratios, large pupae, and less movement. *Aedes albopictus* showed a higher viral RNA titer when energy reserves were lower, while *Cx. pipiens* s.s./*Cx. torrentium* had a higher viral RNA titer when more lipid was stored.

The PCs for *Ae. japonicus* together explained 56.93% of the proportion of variance (Table 2). No infection with JEV was found; therefore, viral RNA titer and infection rate were excluded. Size, energy, and protein content were grouped together for PC1 and were negatively correlated to larval ratio and velocity. For PC2, body contact was negatively correlated with velocity, larval ratio, distance moved, and mortality. This meant that interspecific ratios with lower mobility rates and mortality had a larger pupal size, more body contact, and higher lipid, protein, and glycogen content (Figure 5c, Table 2).

In the corresponding *Cx. p. molestus* strain, 50.48% of the proportion of variance was explained by both PCs (Table 2). For PC1, the distance moved, velocity, cephalothorax area, and protein content were grouped and negatively correlated to larval ratio and development time. The PC2 results indicated that viral RNA titer and velocity were negatively correlated to size and body contact. This indicated that within interspecific ratios, there was fast development, larger pupae, more protein content, greater velocity, and a higher viral RNA titer (Figure 5d).

## 4. Discussion

The present study confirmed that larval interactions between *Ae. albopictus* and *Cx. pipiens* s.s./*Cx. torrentium* and those between *Ae. japonicus* and *Cx. p. molestus* carried over to affect the capability for arboviral infection in the adult stage, at least for *Ae. albopictus* and both *Cx. pipiens* strains. Our findings indicated that all three species were significantly affected by interspecific competition, although out-competition became rarely evident at the levels of the 14 variables tested. Synecological patterns were in general most expressed at often-neglected metabolic and behavioral levels and were particularly pronounced in *Cx. pipiens* at the lower temperature tested, in *Ae. albopictus* at the higher temperature tested, and in *Ae. japonicus* at both temperatures tested.

### 4.1. Aedes albopictus vs. Culex pipiens s.s./Cx. torrentium

Development times for *Ae. albopictus* and *Cx. pipiens* s.s./*Cx. torrentium* were similar at 20 °C; however, at 26 °C, the thermophilic *Ae. albopictus* was faster, especially during interspecific competition, which was also found by Müller, Knautz [17]. Activity was higher in both *Aedes* species compared with *Cx. pipiens* s.s./*Cx. torrentium*. *Aedes* species are known to actively search for their food, browsing on surfaces, while *Cx. pipiens* is a filter feeder hanging near the water surface [33,58,59]. This could imply that the foraging behavior of *Cx. pipiens* is well adapted in scenarios with abundant food, in which they conserve more energy than their competitors [17]. *Aedes albopictus* is known to be the strongest competitor in resource-limiting conditions, due to its active search for food [16,23,25,27,28]; however, it might be less successful compared with *Cx. pipiens* in eutrophic conditions [17,28,30]. In addition, the lipid and glycogen content of *Ae. albopictus* was positively affected by the presence of *Cx. pipiens* s.s./*Cx. torrentium* at 20 °C, whereas at 26 °C, *Ae. albopictus* was negatively affected by its presence. During protein storage, *Ae. albopictus* was always positively affected by the presence of *Cx. pipiens* s.s./*Cx. torrentium* while the latter species remained unaffected.

### 4.2. Aedes japonicus vs. Culex pipiens Bioform molestus

At 20 °C, no influence of interspecific competition was found for larval development time, and at 26 °C, *Cx. p. molestus* developed faster in interspecific competition, outcompeting *Ae. japonicus*. This is in line with Andreadis and Wolfe [31] and Giunti, Becker [60], who observed that *Ae. japonicus* does not tolerate high temperatures and prefers colder habitats. *Culex p. molestus* remained unaffected by *Ae. japonicus* during lipid acquisition; moreover, it even benefitted from its presence during glycogen uptake, indicative of it having more energy reserves available in interspecific combinations. During protein uptake, *Aedes japonicus* was not affected by the presence of *Cx. p. molestus*, but the latter species benefitted from interspecific competition. This observation is in line with Andreadis and Wolfe [31] and with Hardstone and Andreadis [32], who predicted that *Ae. japonicus* would not outcompete *Cx. pipiens*. It is considered a weak larval competitor compared with *Ae. albopictus* [24,33].

### 4.3. Effect of Interspecific Competition on Viral Infection

This study indicates that interspecific larval competition may enhance at least the infection rates and body titers of *Ae. albopictus* and *Cx. pipiens*. Our experiments demonstrated that interspecific competition resulted in larger mosquitoes, a higher arboviral infection rate, and increased arboviral RNA titer. Large mosquitoes benefit from improved longevity, blood feeding, and vector capacity [39]. These are crucial factors to complete the extrinsic incubation period in order to transmit a virus [21,36]. Additionally, Alto, Lounibos [19] and Bevins [21] observed that stressed, larger females attained higher viral titers for SINDV and were more likely to disseminate LACV and develop midgut infection. Conversely, smaller and stressed females may have a weaker immune response [35, 41, 61], potentially enhancing their vector competence. However, their shorter longevity raises uncertainty about whether they can survive long enough to complete the extrinsic incubation period [21,36]. Interspecific larval competition might therefore have an impact on the expression of important factors in the immune system, influencing vector competence.

The calculated RCC revealed that *Ae. albopictus* was subject to competition, which was particularly evident in its reduced lipid intake. This finding suggests a potential increase in sensitivity to viral infection, since lipids are involved in the regulation of the immune response [35,39,40].

The influence of the reduced lipid intake in *Ae. albopictus* was evidenced by the negative correlation with the viral RNA titer of *Ae. albopictus* in the PCA. Additionally, infection rate and viral RNA titer were positively correlated with interspecific competition. Similarly, infection rates in both *Cx. pipiens* strains were higher during interspecific competition. Consistent with previous research by Bevins [21], females subjected to intraspecific treatments were found to be less susceptible to developing LACV infections. These findings suggest that competitive larval interactions may not directly influence DENV replication, but may contribute to a reduction in barriers that impede viral transmission [20].

In this study, we observed that interspecific competition among larvae resulted in larger pupae with elevated infection rates and increased viral RNA titers in the female adult stage. However, interspecifically challenged *Ae. albopictus* had less energy storage and a higher viral RNA titer, while *Cx. pipiens* had a higher viral RNA titer when more lipids were stored. This might be explained by the fact that lipids are essential for flaviviruses to infect cells, replicate, and spread throughout the body. They facilitate the virus’s release from infected cells into new ones. This interaction between lipids and DENV in *Ae. albopictus* and *Ae. aegypti* has been well studied by Perera, Riley [62], Chotiwan, Andre [63], and Koh, Islam [64].

### 4.4. Large-Scale Implications

This study demonstrates a combination of the factors mentioned above. After interspecific competition, mosquitoes were larger and had a higher infection rate, as well as a higher viral RNA titer. This indicates that interspecific larval competition may enhance the vector competence of *Ae. albopictus* and *Cx. pipiens*. Large mosquitoes benefit from improved longevity, blood feeding, and vector capacity [39]. These factors are essential for completing the extrinsic incubation period required for virus transmission [21,36]. Additionally, these larger females attain higher viral titers [19,21]. On the other hand, smaller and stressed females may exhibit weaker immune response [35,41,61], which could increase their vector competence. Nonetheless, their reduced lifespan leads to uncertainty about whether they can survive through the entire extrinsic incubation period [21,36].

### 4.5. Limitations of the Study

No virus transmission rate was investigated in this study, due to the large number of experimental treatments and species tested. However, the relevance of carry-over effects from larval to adult stage for the vector competence of mosquitoes merits further research. Additionally, the patterns found in laboratory environments may be different than those in the field [21]. Additional factors shaping larval microhabitats need to be taken into account, such as temperature, food source, water quality, larval density, species composition, and physical characteristics. Moreover, behavioral and immune responses of the resulting adult mosquitoes of the tested bioforms and strains, and finally, microbiome and viral doses, need to be further considered. Here, we tested two different bioforms of *Cx. pipiens*, which could have had an effect on the results and merits further research. A very promising avenue of research is the effect of qualitative and quantitative lipid accumulation during larval stage on arboviral vector competence, to better understand the causative links in semi-aquatic environments. We observed an opposite pattern between *Ae. albopictus* and *Cx. pipiens* related to their lipid storage and infection rate. Additionally, we found that interspecific combinations developed faster and had larger pupae. This paradoxical observation requires further research.

## 5. Conclusions

This study provides an in-depth insight into the larval competition between *Ae. albopictus*, *Ae. japonicus*, and *Cx. pipiens*, and the associated carry-over effect of synecological patterns to higher arboviral infection during their adult stage. During interspecific competition, all species developed faster and had larger pupae and more protein storage. The competition between *Ae. albopictus* and *Cx. pipiens* varied, but the results indicate that *Ae. albopictus* is the better competitor in resource-limited habitats while *Cx. pipiens* thrives in eutrophic situations. *Aedes japonicus* was always a weaker competitor compared with *Cx. pipiens*. *Aedes albopictus* and *Cx. pipiens* were more susceptible to arboviral infection after interspecific competition, and storage of lipids was lower in *Ae. albopictus* while it was higher in *Cx. pipiens*. This could suggest that lipids are involved in both the regulation of the immune response and in virus infection in the mosquito. No infections with JEV nor any natural arbovirus infections were found in *Ae. japonicus*.

## Figures and Tables

**Table 1:** Infection rates (IR), viral RNA copy number/body (mean log10 RNA copies/specimen), and statistical significance for *Ae. albopictus*, *Cx. pipiens* s.s./*Cx. torrentium*, and *Cx. p. molestus*.

Virus	Mosquito Species	Species Ratio	Dpi	n	IR [%]	Viral RNA Copy Number/Body [log10]	Significance
CHIKV	*Ae. albopictus*	30 *Aedes* intra	7	20	100	8.99 ± 1.58	0.04
		20 *Aedes* inter		23	100	9.36 ± 1.81	
		10 *Aedes* inter		14	100	9.41 ± 0.94	ns
WNV	*Cx. pipiens* s.s./*Cx. torrentium*	30 *Culex* intra	14	42	54.76	5.39 ± 1.7	ns
		20 *Culex* inter		34	50	5.07 ± 1.59	
		10 *Culex* inter		9	66.67	5.69 ± 1.78	
	*Cx. p. molestus*	30 *Culex* intra	14	91	43.96	6.61 ± 2.1	ns
		20 *Culex* inter		64	54.69	6.34 ± 2.05	
		10 *Culex* inter		27	59.26	5.78 ± 1.8	

**Table 2:** Overview of the loadings per variable for each species and principal component (PC). The six highest variables (development time, larval ratio, mortality, larval ratio, cephalothorax length (CL), abdominal length (AL), abdominal width (AW), lipid (L), protein (P), glycogen (G), mortality, total distance moved, velocity, body contact, viral RNA body titer (BT), and infection rate (IR)) per species and principal component are included in this overview.

PC1							
*Ae. albopictus*		*Cx. pipiens* s.s./*Cx. torrentium*		*Ae. japonicus*		*Cx. p. molestus*	
Development	0.78	Development	−0.51	L	−0.58	Distance	0.81
Ratio	0.77	Mortality	−0.58	G	−0.76	Velocity	0.77
AL	−0.71	Body contact	−0.67	P	−0.82	CT	0.63
AW	−0.73	P	−0.69	AW	−0.86	P	0.60
CT	−0.79	Distance	−0.70	AL	−0.88	Development	−0.83
P	−0.81	Velocity	−0.74	CT	−0.92	Ratio	−0.84
**PC2**							
***Ae. albopictus***		***Cx. pipiens*** **s.s./*Cx. torrentium***		***Ae. japonicus***		***Cx. p. molestus***	
BT	0.58	CT	0.57	Body contact	0.61	AW	0.68
Ratio	−0.59	Ratio	0.55	L	−0.27	CT	0.63
Velocity	−0.65	BT	0.50	Mortality	−0.59	Body contact	0.62
Distance	−0.65	L	0.50	Distance	−0.68	AL	0.62
L	−0.68	Velocity	−0.63	Ratio	−0.81	BT	−0.38
G	−0.80	Distance	−0.66	Velocity	−0.88	Velocity	−0.56

## Data Availability

Data supporting the conclusions of this article are included within the article. The datasets generated and analyzed during the current study are available in the Zenodo repository, DOI number 10.5281/zenodo.12820697.

## Supplementary Materials

The following supporting information can be downloaded at: https://www.mdpi.com/article/10.3390/v16081202/s1, Figure S1: Mortality under intraspecific competition; Figure S2: Mortality under interspecific competition; Figure S3: Development time during interspecific competition between the different species. Figure S4: Pupal size of *Ae. albopictus*, *Ae. japonicus*, and *Cx. pipiens* at 20 °C and 26 °C under interspecific competition. Table S1: Coefficient of variation of the pupal growth of *Ae. albopictus*, *Ae. japonicus*, *Cx. pipiens s.s*./*Cx. torrentium*, and *Cx. p. molestus*; Figure S5: Total distance moved, velocity and body contact measured during two minutes for different larval ratios; Figure S6: Size corrected lipid, glycogen and protein content per pupae from interspecific competition ratios at 20 °C and 26 °C.

## Abbreviations

AL: abdominal length; ANOVA: analysis of variance; AW: abdominal width; BNITM: Bernhard Nocht Institute for Tropical Medicine; BT: body titer; CHIKV: chikungunya virus; CT: cephalothorax area; DENV: dengue virus; G: glycogen; IR: infection rate; ITM: Institute of Tropical Medicine; JEV: Japanese encephalitis virus; L: lipid; LACV: La Crosse encephalitis virus; P: protein; PCA: principal component analysis; WNV: West Nile virus.
