# Supplementary material for: Larval Competition between Aedes and Culex Mosquitoes Carries over to Higher Arboviral Infection during Their Adult Stage
Source: Viruses. 2024 Jul 26;16(8):1202. doi: 10.3390/v16081202 (PMC11359221; doi:10.3390/v16081202)
Supplement: Supplementary file 1 [file viruses-16-01202-s001.zip › viruses-3099497-supplementary.pdf]

Supplementary files

Supplementary file S1

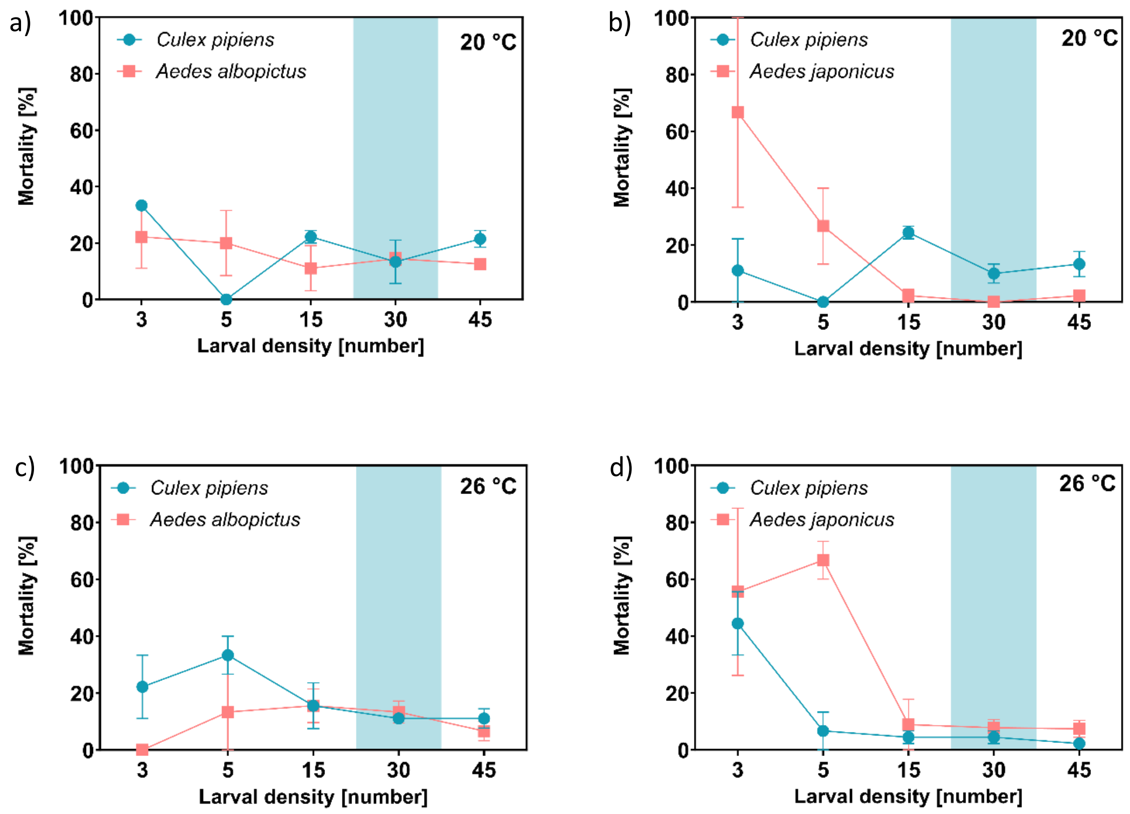

Figure S1 Mortality at 20°C in a) *Ae. albopictus* vs *Cx. pipiens* s.s./*Cx. torrentium* and b) *Ae. japonicus* vs *Cx. p. molestus*, and at 26°C in c) *Ae. albopictus* vs *Cx. pipiens* s.s./*Cx. torrentium* and d) *Ae. japonicus* vs *Cx. p. molestus* under intraspecific competition to test which larval density is adequate for interspecific competition ratios. In blue is the percentage of mortality for a density of 30 larvae.

Statistics: Data was not normally distributed therefore a Friedman test was conducted, yielding non-significant results.

Supplementary file S2

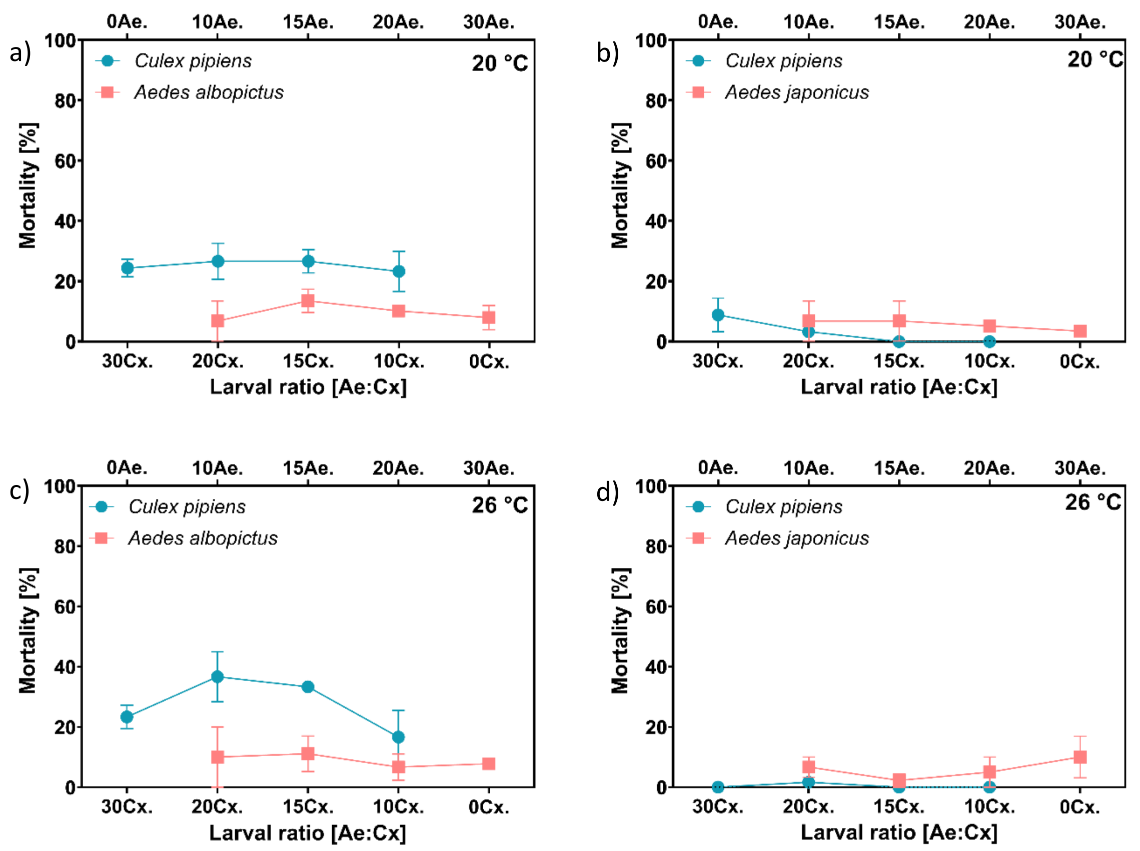

Figure S2 Mortality at 20°C in a) *Ae. albopictus* vs *Cx. pipiens* s.s./*Cx. torrentium* and b) *Ae. japonicus* vs *Cx. p. molestus*, and at 26°C in c) *Ae. albopictus* vs *Cx. pipiens* s.s./*Cx. torrentium* and d) *Ae. japonicus* vs *Cx. p. molestus* under interspecific competition.

Statistics: Data was not normally distributed, therefore a Friedman test was conducted (p = 0.01). Multiple comparisons were not significant. Mortality was highest in interspecific combinations for the *Ae. albopictus* x *Cx. pipiens* s.s./*Cx. torrentium* combination while for *Ae. japonicus* x *Cx. p. molestus* mortality was higher interspecific at 20°C and intraspecific at 26°C.

Supplementary file S3

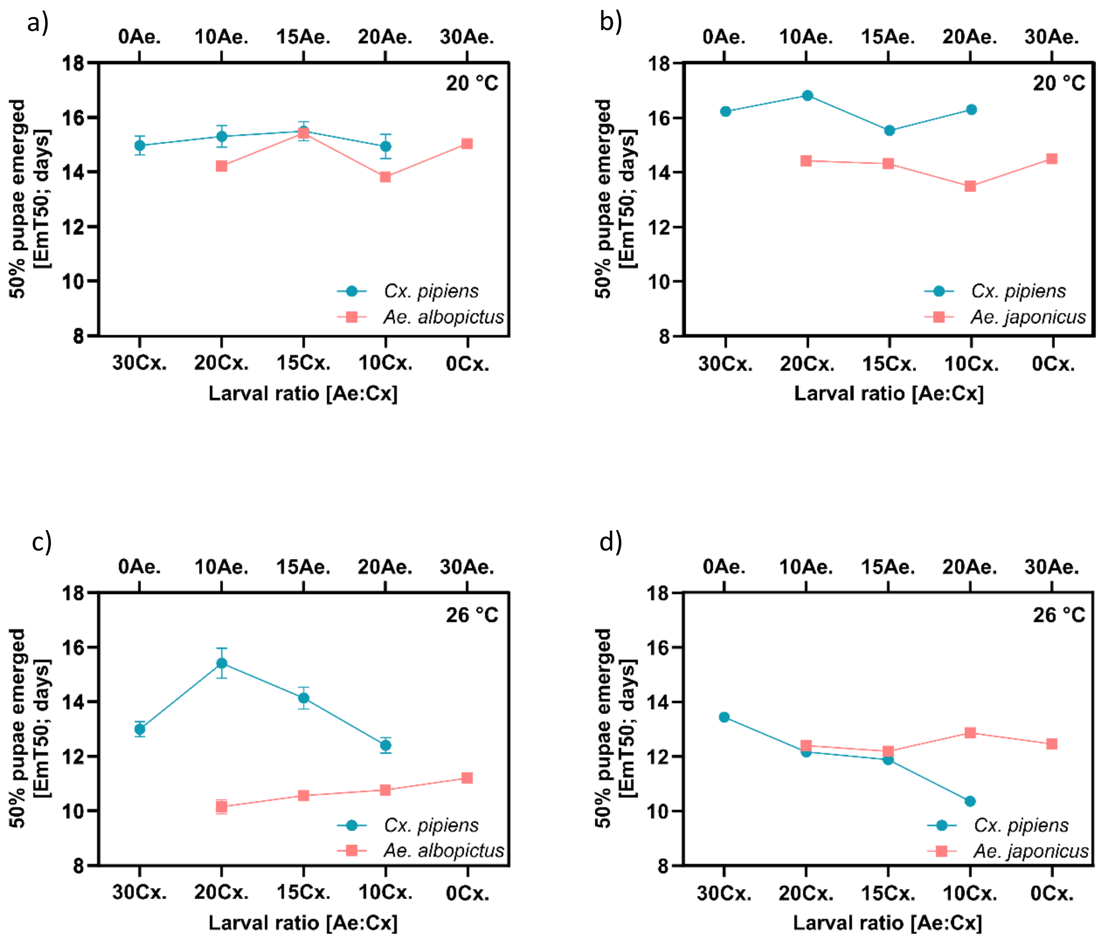

Figure S3 Development time during interspecific competition between the different species. a) competition between *Ae. albopictus* and *Cx. pipiens* s.s./*Cx. torrentium* at 20°C, b) *Ae. japonicus* and *Cx. p. molestus* at 20°C, c) *Ae. albopictus* and *Cx. pipiens* s.s./*Cx. torrentium* at 26°C, d) *Ae. japonicus* and *Cx. p. molestus* at 26°C.

Statistics: A two-way ANOVA was conducted to compare the development time for 50 % for the pupae to emerge. For the combination *Ae. albopictus* vs *Cx. pipiens* s.s./*Cx. torrentium* at 20°C, *Species* ($F = 8.816$; $p = 0.007$), *Larval ratio* ($F = 4.691$; $p = 0.01$), and its *Interaction* ($F = 3.294$; $p = 0.04$) are significantly different. Tukey's multiple comparisons test showed a difference between interspecific 20 *Cx. pipiens* vs. 20 *Ae. albopictus* ($p = 0.01$), 20 *Ae. albopictus* vs. 15 *Cx. pipiens* ($p = 0.005$), 20 *Ae. albopictus* vs. 15 *Ae. albopictus* ($p = 0.008$), 15 *Cx. pipiens* vs. 10 *Ae. albopictus* ($p = 0.05$) (Supplementary file 3 Figure S3a). At 26°C, the *Larval ratio* ($F = 4.545$; $p = 0.01$), *Species* ($F = 77.40$; $p < 0.001$), and its *Interaction* ($F = 3.298$; $p = 0.03$) are significantly different. Tukey's multiple comparisons test showed significant differences for 30 *Cx. pipiens* vs. 20 *Cx. pipiens* ($p = 0.04$), 20 *Cx. pipiens* vs. 10 *Cx. pipiens* ($p = 0.005$), and 20 *Cx. pipiens* vs. 20 *Ae. albopictus* ($p < 0.001$) (Supplementary file 3 Figure S3c).

The combination *Ae. japonicus* vs *Cx. p. molestus* at 20°C showed significant differences for factors *Species* ($F = 139.3$; $p < 0.001$) and *Interaction between species and larval ratio* ($F = 6.569$; $p =$

0.002). Tukey's multiple comparisons test showed significant differences between 30 *Cx. pipiens* vs. 30 *Ae. japonicus* ($P < 0.001$), 20 *Cx. pipiens* vs. 20 *Ae. japonicus* ($p < 0.001$), 15 *Cx. pipiens* vs. 15 *Ae. japonicus* ($p = 0.03$), 10 *Cx. pipiens* vs. 10 *Ae. japonicus* ($p < 0.001$), 20 *Cx. pipiens* vs. 15 *Cx. pipiens* ($p = 0.02$) (Supplementary file 3 Figure S3b). At 26°C, the *Interaction between species and larval ratio* (F = 3.651 ; $p = 0.003$) and *Larval ratio* (F = 4.287 ; $p = 0.01$) were significantly different. Tukey's multiple comparisons test showed a difference between the 30 intraspecific and 10 interspecific *Cx. pipiens* ($p = 0.002$) and 10 interspecific *Cx. pipiens* with 20 interspecific *Ae. japonicus* ($p = 0.02$) (Supplementary file 3 Figure S3d).

Supplementary file S4

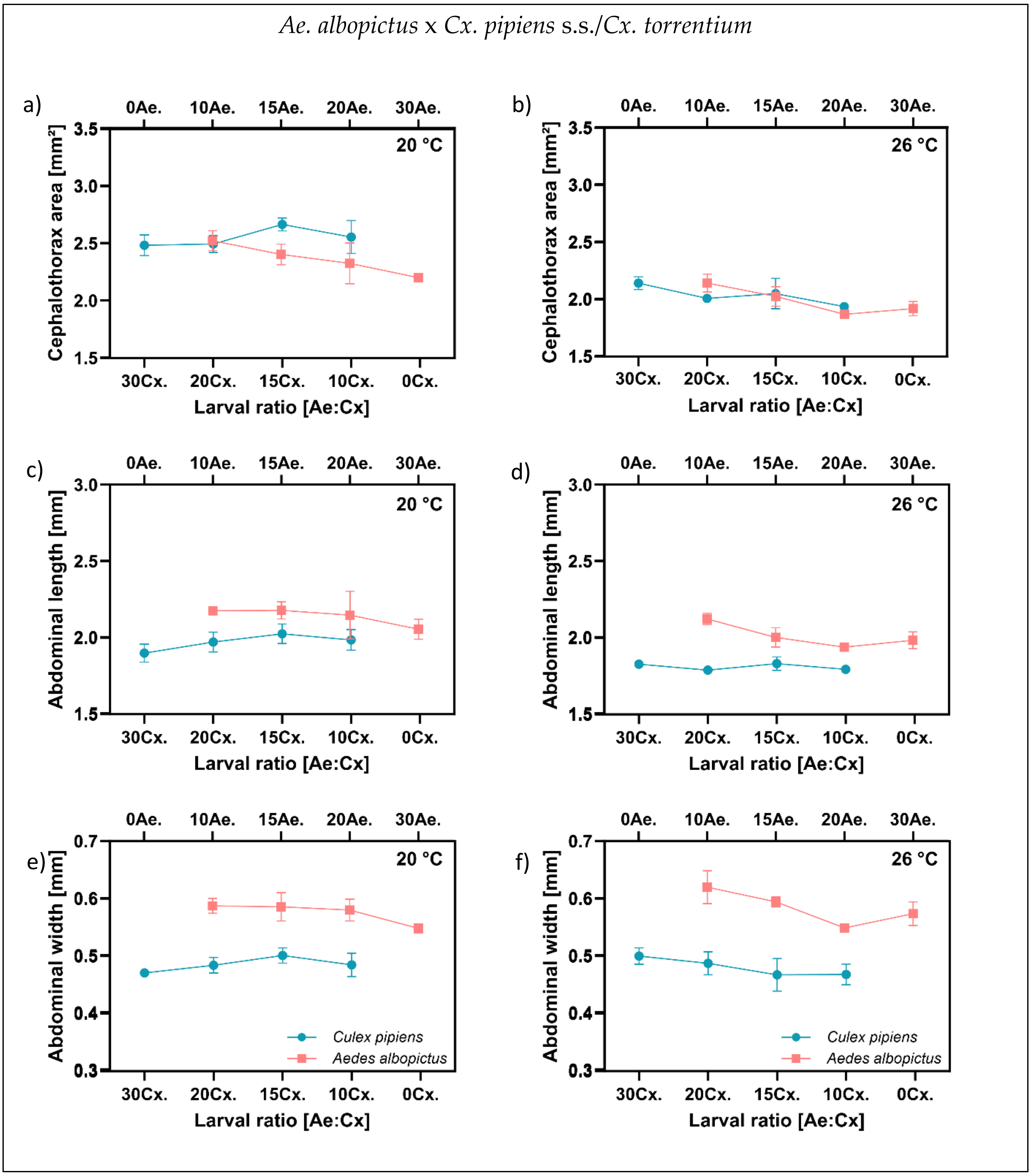

Ae. japonicus x *Cx. pipiens bioform molestus*

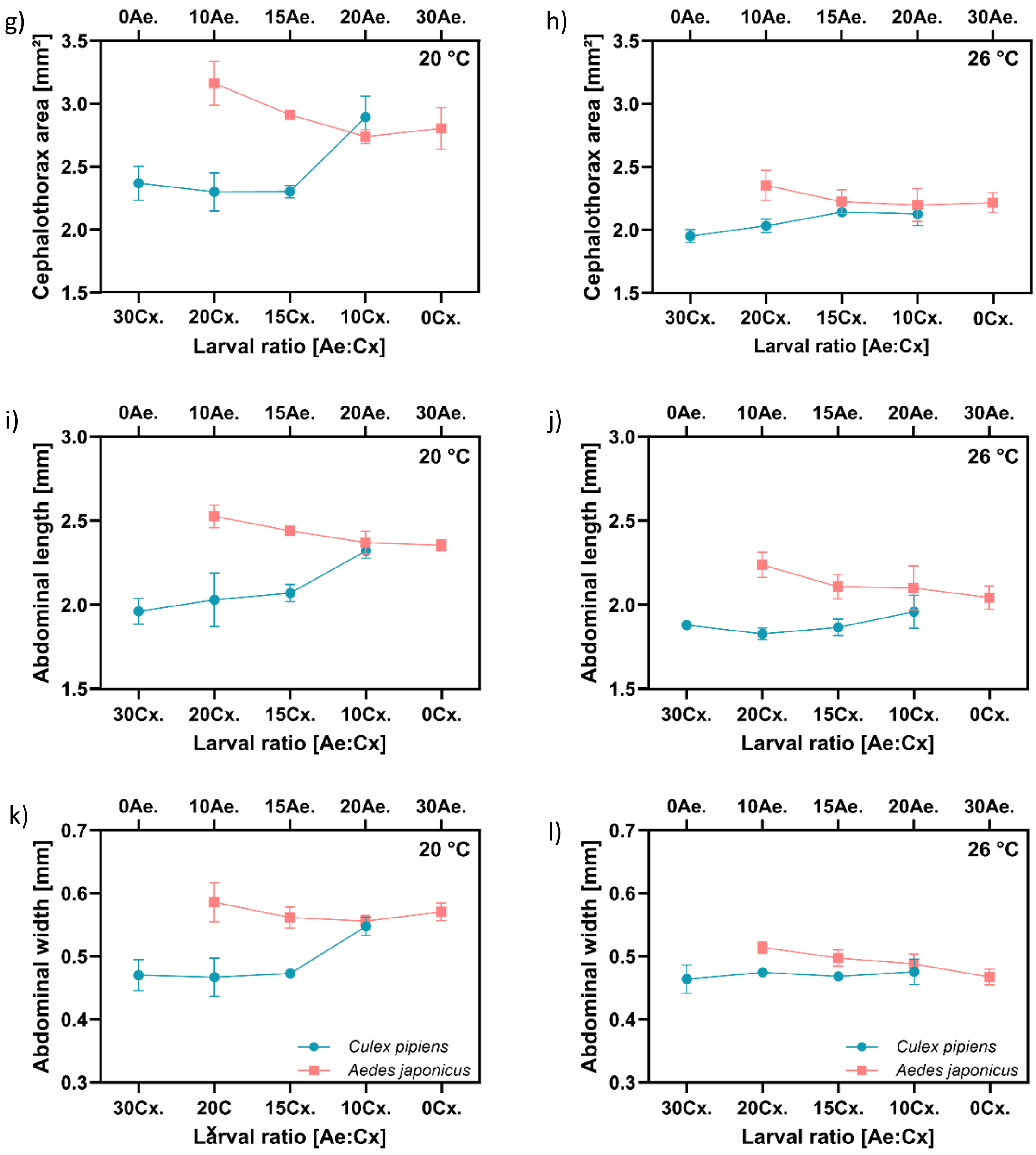

Figure S4 Pupal size of *Ae. albopictus*, *Ae. japonicus*, and *Cx. pipiens* at 20°C and 26°C under interspecific competition.

Statistics: A two-way ANOVA was conducted to compare the pupal size for *Ae. albopictus*, *Ae. japonicus* and *Cx. pipiens* at 20°C and 26°C under interspecific competition. For the area of the cephalothorax at 20°C, the factors *Species* (F = 15.70 ; $p < 0.001$) and *Species ratio* (F = 6.804 ; p = 0.001) are significantly different. Tukey's multiple comparisons showed significant differences within *Culex pipiens* (from *Ae. japonicus* ratio) when comparing 30 vs 10 (p = 0.01), 20 vs 10 (p = 0.005) and 15 vs 10 (p = 0.005) *Cx. pipiens* ratios. Between the species, there was a significant difference between 20 *Ae. japonicus* vs *20 Cx. pipiens* (p = 0.05), and 15 *Ae. japonicus* vs 15 *Cx.*

pipiens (Supplementary file 4 Figure S4a-b). At 26°C, only the factor species was significantly different (F = 7.525 ; p < 0.001) (Supplementary file 4 Figure S4c-d).

The abdominal length at 20°C was significantly different for factors *Species* (F = 22.02 ; p < 0.001) and *Species ratio* (F = 3.817 ; *p* = 0.02). Tukey's multiple comparisons showed significant difference for 30 vs 10 *Cx. pipiens* (*p* = 0.01), and for all densities between *Ae. japonicus* vs *Cx. pipiens* (both ratios). At 26°C, only *Species* (F = 19.70 ; *p* < 0.001) was significantly different. The multiple comparisons showed a significant difference for 20 *Ae. japonicus* vs. 20 *Cx. pipiens* (*p* = 0.02), for 15 *Ae. japonicus* vs. 15 *Cx. pipiens* (*p* = 0.04), for 10 Ae. albopictus vs 10 *Cx. pipiens* (*p* = 0.003), and for *Ae. japonicus* vs. 10 *Cx. pipiens* (*p* = 0.01).

The abdominal width at 20°C shows a significant difference for factors *Species* (F = 27.67 ; *p* < 0.001) and *Species ratio* (F = 2.928 ; *p* = 0.05). The multiple comparisons is significantly different for *Cx. p. molestus* in 30 vs 10 (*p* = 0.03), 20 vs 10 (*p* = 0.02), and 15 vs 10 (*p* = 0.03) and all species ratios. At 26°C, *Species* was significantly different (F = 36.02 ; *p* < 0.001). Tukey's multiple comparisons test showed significant differences for *Ae. albopictus* between 20 vs 10 larval densities (*p* = 0.03), 30 *Ae. albopictus* vs 30 *Cx. pipiens* (*p* = 0.03), 15 *Ae. albopictus* vs 15 *Cx. pipiens* (*p* < 0.001), and 10 *Ae. albopictus* vs 10 *Cx. pipiens* (*p* < 0.001).

Supplementary file S5

Tabel S1: Coefficient of variation of the pupal growth of *Ae. albopictus*, *Ae. japonicus, Cx. pipiens* s.s./*Cx. torrentium*, and *Cx. p. molestus.*

Species	T [°C]	Cephalothorax area [mm²]	Abdominal length [mm]	abdominal width [mm]
Ae. albopictus	20	0.35	0.19	0.06
Ae. albopictus	26	0.28	0.18	0.07
Ae. japonicus	20	0.48	0.26	0.07
Ae. japonicus	26	0.33	0.22	0.05
Cx. pipiens s.s./*Cx. torrentium*	20	0.30	0.17	0.05
Cx. pipiens s.s./*Cx. torrentium*	26	0.24	0.16	0.06
Cx. p. molestus	20	0.44	0.26	0.06
Cx. p. molestus	26	0.22	0.18	0.05

Supplementary file S6

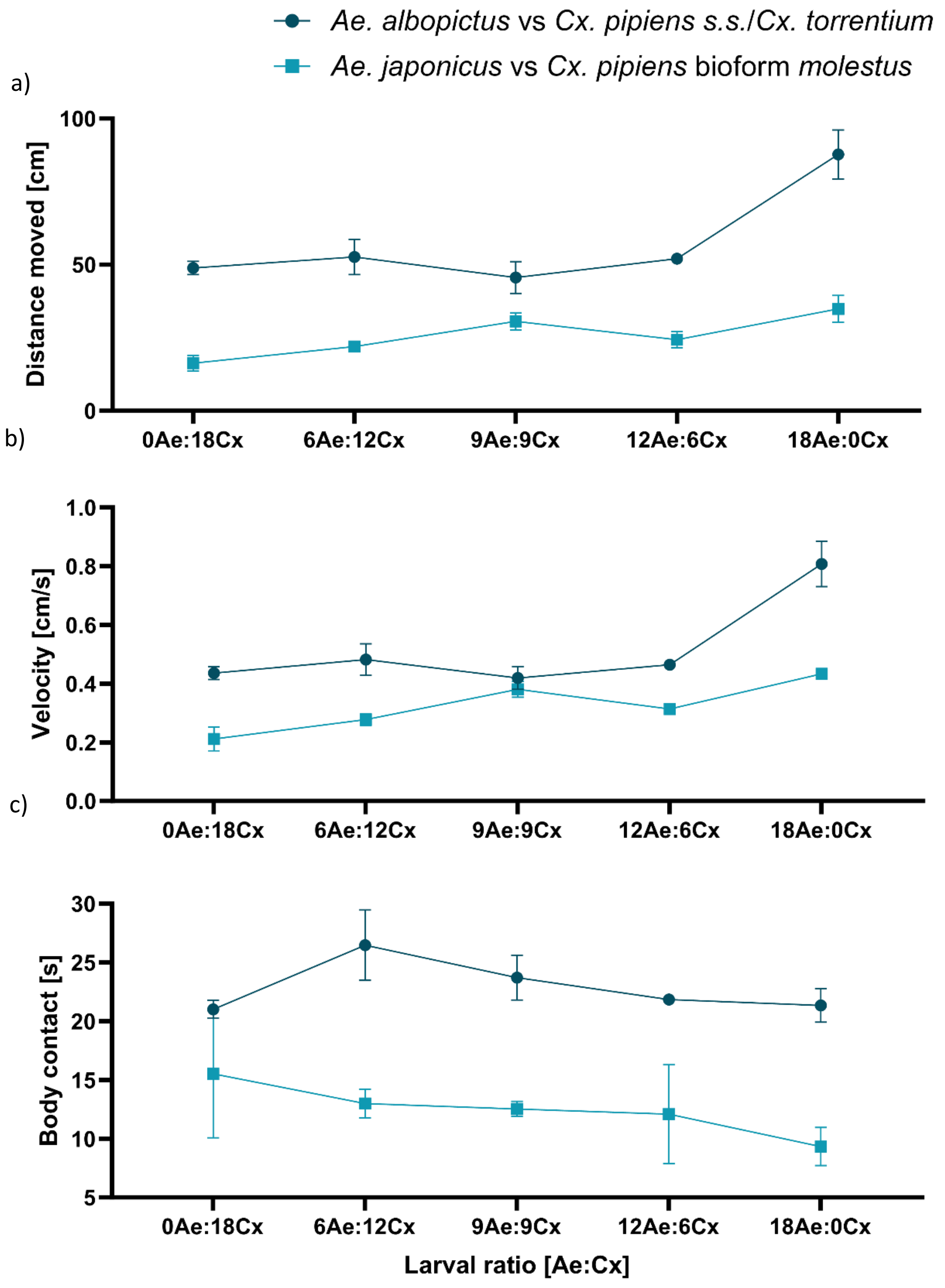

Figure S5 Total distance moved, velocity and body contact measured during two minutes for different larval ratios in the *Ae. albopictus* and *Cx. pipiens* s.s./*Cx. torrentium*, and *Ae. japonicus* and *Cx. p. molestus* combinations.

Statistics: Data of the distance moved was sine transformed to assume normal distribution and a two-way ANOVA showed no significant differences between the species combinations and ratios. Data on the velocity violated normality, therefore multiple Mann-Whitney tests

were conducted, showing no significant differences. For body contact a two-way ANOVA was conducted, showing a significant difference for the factor *Species combination* ($F = 40.17$; $p < 0.001$). Tukey's multiple comparisons test showed significant differences between both species combinations for the ratios 6A:12C ($p = 0.001$), 9A:9C ($p = 0.006$), 12A:6C ($p = 0.01$), and 18A:0C ($p = 0.004$).

Supplementary file S7

Ae. albopictus x *Cx. pipiens* s.s./*Cx. torrentium*

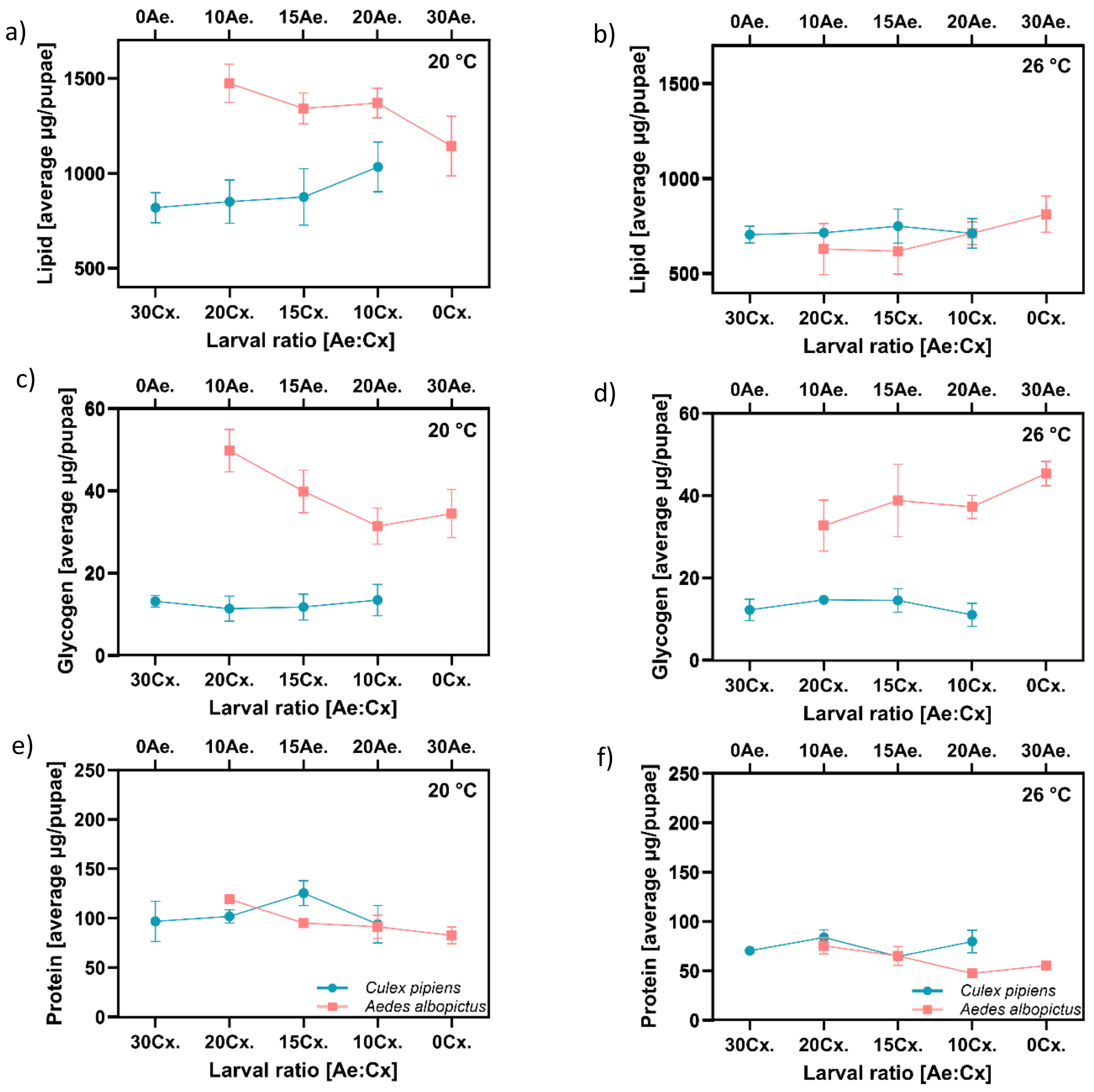

Ae. japonicus x *Cx. pipiens bioform molestus*

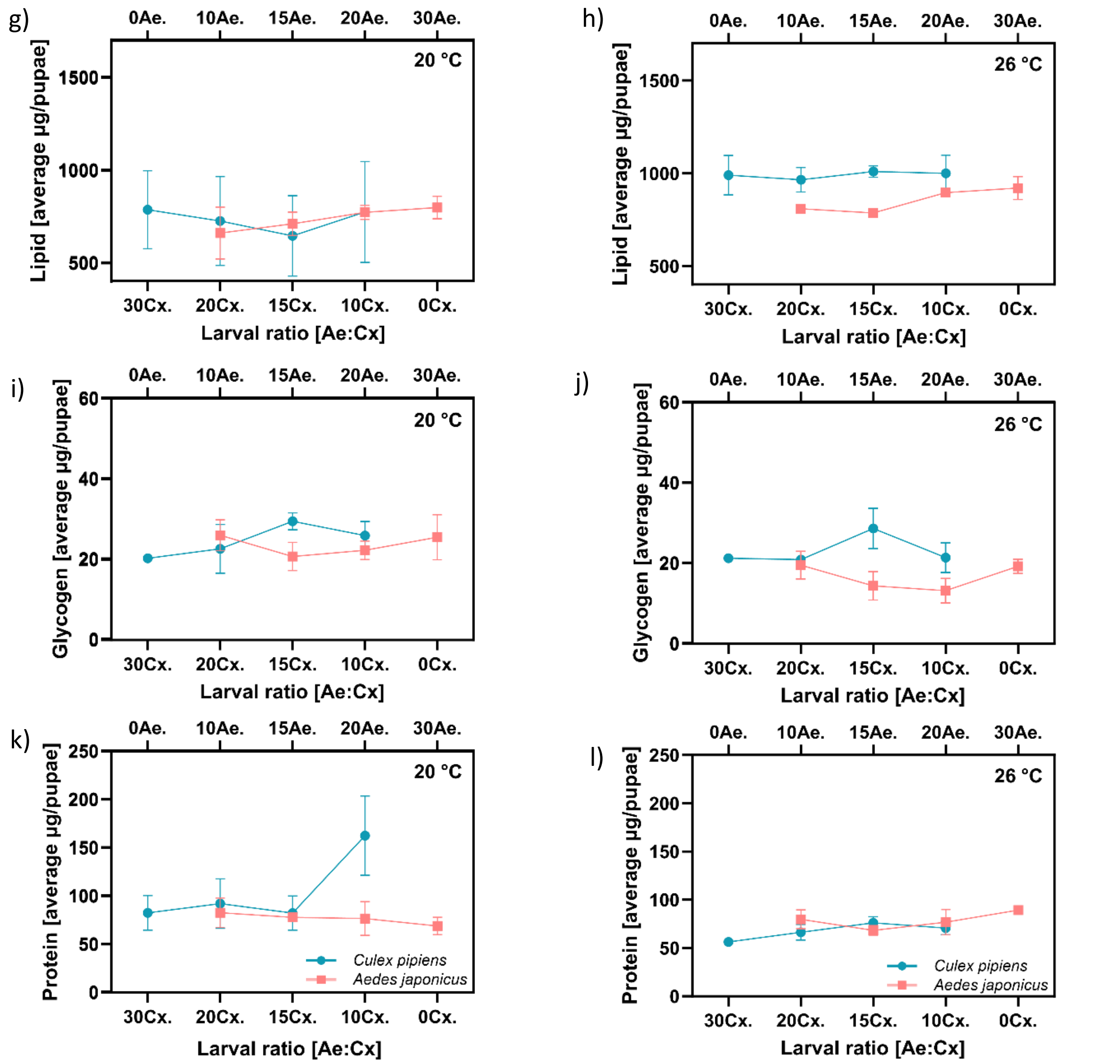

Figure S6 Size corrected lipid, glycogen and protein content per pupae from interspecific competition ratios at 20°C and 26°C.

Statistics: For the lipids, a two-way ANOVA revealed significant difference for the factor *Species and temperature* (F = 33.21 ; p < 0.001) in the *Ae. albopictus* x *Cx. pipiens* s.s./*Cx. torrentium* combination. Tukey's multiple comparisons test showed significant differences for 10 *Cx. pipiens* vs 10 *Ae. albopictus* (p = 0.02), 15 *Cx. pipiens* vs 15 *Ae. albopictus* (p = 0.02), and 20 *Cx. pipiens* vs 20 *Ae. albopictus* at 20°C (p = 0.006). *Ae. albopictus* has very high values at 20°C, with less lipids in intraspecific competition. At higher temperature, the lipid content drops, but an opposite trend is observed with more lipids in intraspecific competition. For *Cx. pipiens* the difference between 20 and 26°C was much smaller, but at higher temperatures the lipid content was also lower. The factor *Species and temperature* (F = 3.006 ; p = 0.05) was also significantly different in the *Ae. japonicus* x *Cx. p. molestus* combination, but no differences were found in the Tukey multiple comparisons. They showed higher lipid content at higher temperatures,

with highest differences in *Cx. pipiens*. *Aedes japonicus* showed higher lipid contents intraspecifically compared to interspecific competition.

The glycogen content in the *Ae. albopictus* x *Cx. pipiens* s.s./*Cx. torrentium* combination showed a significant difference for the factor *Species and temperature* ($F = 48.28$; $p < 0.001$). Tukey's multiple comparisons test showed significant differences for 10 *Cx. pipiens* vs 10 *Ae. albopictus* at 20 ($p < 0.01$) and 26°C ($p = 0.007$), 15 *Cx. pipiens* vs 15 *Ae. albopictus* at 20 ($p < 0.01$) and 26°C ($p = 0.002$), 20 *Cx. pipiens* vs 20 *Ae. albopictus* at 20 ($p = 0.01$) and 26°C ($p = 0.005$), 30 *Cx. pipiens* vs 30 *Ae. albopictus* at 20 ($p = 0.007$) and 26°C ($p < 0.01$), and between 10 vs 20 *Ae. albopictus* at 20°C ($p = 0.03$). The glycogen content was always higher in *Ae. albopictus* compared to *Cx. pipiens*, with likewise to lipids a higher glycogen content intraspecifically at 26°C and lower glycogen content intraspecifically at 20°C for *Ae. albopictus*. In *Cx. pipiens*, variation between the two temperatures and competition was limited. The *Ae. japonicus* x *Cx. p. molestus* combination was sine transformed to assume normality. A two-way ANOVA showed no significant differences in glycogen content. The variation in glycogen was minimal for *Cx. pipiens*. *Aedes japonicus* exhibited a lower glycogen content at 26°C compared to 20°C.

The protein content in the *Ae. albopictus* x *Cx. pipiens* s.s./*Cx. torrentium* combination showed a significant difference for the factor *Species and temperature* ($F = 15.99$; $p < 0.001$). Tukey's multiple comparisons test showed no significant differences. *Aedes albopictus* always had higher protein content in interspecific competition compared to intraspecific competition. *Culex pipiens* had some variety in interspecific competition ratios. Both species exhibit lower protein contents at higher temperatures. Data on the *Ae. japonicus* x *Cx. p. molestus* combination was sine transformed to assume normality. A two-way ANOVA showed no significant differences in protein content. Variation of protein content in *Ae. japonicus* was limited. *Culex pipiens* showed a higher protein content interspecifically compared to intraspecific competition.
